# Trends in solitary plasmacytoma, extramedullary plasmacytoma, and plasma cell myeloma incidence and myeloma mortality by racial‐ethnic group, United States 2003‐2016

**DOI:** 10.1002/cam4.3444

**Published:** 2020-12-03

**Authors:** Taylor D. Ellington, S. Jane Henley, Reda J. Wilson, Manxia Wu, Lisa C. Richardson

**Affiliations:** ^1^ Division of Cancer Prevention and Control, National Center for Chronic Disease Prevention and Health Promotion Centers for Disease Control and Prevention Atlanta GA USA

**Keywords:** cancer surveillance, epidemiology, myeloma

## Abstract

Plasma cell myeloma (also called multiple myeloma), solitary plasmacytoma, and extramedullary plasmacytoma are primarily diseases of the elderly. Evidence suggests an association between excess body weight and multiple myeloma. Few population‐based studies have examined incidence and mortality of each site in one study. We analyzed incidence and death rates by site (solitary plasmacytoma, extramedullary plasmacytoma, and multiple myeloma) by gender, age, race/ethnicity, and rural‐urban status among adult males and females (aged 20 years or older) in the United States during 2003‐2016. Trends were characterized as average annual percentage change (AAPC) in rates. During 2003‐2016, overall incidence rates among adults were 0.45 for solitary plasmacytoma, 0.09 for extramedullary plasmacytoma, and 8.47 for multiple myeloma per 100,000 persons. Incidence rates for multiple myeloma increased during 2003‐2016 among non‐Hispanic whites (AAPC = 1.78%) and non‐Hispanic blacks (2.98%) 20‐49 years of age; non‐Hispanic whites (1.17%) and non‐Hispanic blacks (1.24%) 50‐59 years of age; and whites non‐Hispanic (0.91%), and non‐Hispanic blacks (0.96%). During 2003‐2016 overall myeloma (extramedullary plasmacytoma and multiple myeloma) death rates among adults was 4.77 per 100,00 persons. Myeloma death rates decreased during 2003‐2016 among non‐Hispanic white (AAPC = −1.23%) and Hispanic (−1.34%) women; and non‐Hispanic white (−0.74%), non‐Hispanic American Indian/Alaska Native (−3.05%) men. The US population is projected to become older and will have a larger proportion of persons who have had an earlier and longer exposure to excess body weight. The potential impact of these population changes on myeloma incidence and mortality can be monitored with high‐quality cancer surveillance data.

## Graphical abstract

Plasma cell myeloma (also called multiple myeloma), solitary plasmacytoma, and extramedullary plasmacytoma are primarily diseases of the elderly. Few population‐based studies have examined incidence and mortality of each site in one study. We analyzed incidence and death rates by site (solitary plasmacytoma, extramedullary plasmacytoma, and multiple myeloma) by gender, age, race/ethnicity, and rural‐urban status among adult males and females (aged 20 years or older) in the United States during 2003‐2016.

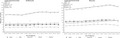

## 1 INTRODUCTION

Plasma cell myeloma, also called multiple myeloma, is a clonal plasma cell proliferative disorder and is the second most common hematologic malignancy, after non‐Hodgkin lymphoma in the United States.^1^, ^2^ In rare cases, plasma cell proliferation occurs in the form of a solitary lesion and can be found in bone (solitary plasmacytoma) or soft tissue (extramedullary plasmacytoma).^3^

Multiple myeloma cases are often preceded by monoclonal gammopathy of undetermined significance.^4^ Recent studies have found associations between excess body weight and multiple myeloma.^5^ Excess body weight during midlife was associated in one study with an increased risk of progression from monoclonal gammopathy of undetermined significance/light‐chain monoclonal gammopathy of undetermined significance to multiple myeloma later in life.^6^

Multiple myeloma is primarily a disease of the elderly with a median age of onset of 74 years.^7^ However, from 1995 to 2014, incidence rates of multiple myeloma increased in younger adults 25‐49 years of age.^8^ Like multiple myeloma, incidence rates for solitary and extramedullary plasmacytoma rise exponentially with advancing age.^9^, ^10^

Few population‐based studies have examined trends in incidence of solitary plasmacytoma, extramedullary plasmacytoma, and multiple myeloma in one study.^11^, ^12^ Previous population‐based studies examined incidence and survival of individuals diagnosed with solitary plasmacytoma, extramedullary plasmacytoma, and multiple myeloma with a focus on racial disparities; and incidence rates were found to be higher among blacks compared to whites.^11^, ^12^, ^13^

To further the understanding of recent rates of solitary plasmacytoma, extramedullary plasmacytoma, and multiple myeloma and deaths attributed to myeloma (extramedullary plasmacytoma and multiple myeloma), we used data from the US Cancer Statistics (USCS), covering the entire US population, to examine trends in incidence and death rates during 2003‐2016. We examined how rates and trends varied by race/ethnicity, gender, age, and rural‐urban status.

## 2 METHODS

### 2.1 Data sources

#### 2.1.1 Cancer incidence data

Population‐based incidence data are from the USCS, which combines the Centers for Disease Control and Prevention's (CDC’s) National Program of Cancer Registries (NPCR) and the National Cancer Institute's surveillance, epidemiology and End Results (SEER) Program datasets.^14^ This dataset includes cancer incidence data from central cancer registries reported to NPCR or SEER from all states and the District of Columbia. Data about all new diagnoses of cancer from patient records at medical facilities such as hospitals, physicians' offices, therapeutic radiation facilities, freestanding surgical centers, and pathology laboratories are reported to central cancer registries, which collate these data and use state vital records to collect information about any cancer deaths that were not reported as cases. Incidence data met USCS publication criteria, covering 100% of the US population during 2003‐2016.

We selected malignant cases of solitary and extramedullary plasmacytoma, and multiple myeloma identified using *International Classification of Diseases for Oncology, Third Edition* (ICD‐O‐3) diagnosed during 2003‐2016.^15^ We restricted morphology codes to include 9731/3 (solitary plasmacytoma), 9734/3 (extramedullary plasmacytoma), and 9732/3 (multiple myeloma). Most cases (87.54%) were microscopically confirmed, 2.24% were confirmed with a laboratory test, 1.61% were confirmed with radiography, 2.05% were confirmed clinically, and type of confirmation was unknown for 6.55%; all were included in this analysis.

#### 2.1.2 Mortality data

Mortality data during 2003‐2016 are from CDC’s National Center for Health Statistics’ National Vital Statistics System. Although more recent mortality data are available, we used data during 2003‐2016 to be consistent with the most recent incidence data available.^16^ We defined myeloma deaths as those with *International Statistical Classification of Diseases and Related Health Problems, 10th Revision* (ICD‐10) codes C90.0 (multiple myeloma) or C90.2 (extramedullary plasmacytoma) as the underlying cause of death.^17^ Almost all (99%) myeloma deaths were multiple myeloma.

#### 2.1.3 Population

Because previous studies found that multiple myeloma is primarily a disease of older age, we restricted this analysis to adult males and females aged 20 years or older.^8^ Age‐specific analyses used these grouped categories: 20‐49, 50‐59, 60‐69, 70‐79, and ≥80 years.

#### 2.1.4 Race and ethnicity

Data were analyzed for five mutually exclusively racial/ethnic groups: non‐Hispanic whites, non‐Hispanic blacks, non‐Hispanic American Indian/Alaska Native, non‐Hispanic Asian/Pacific Islander, and Hispanic. Hispanic ethnicity and race data were collected separately and combined in this analysis.

#### 2.1.5 Rural‐urban status

The US Department of Agriculture Economic Research Service 2013 vintage rural‐urban continuum classification scheme was used to categorize county of residence at diagnosis as metropolitan (rural‐urban continuum codes 1‐3) or nonmetropolitan (rural‐urban continuum codes 4‐9). The mortality dataset included data grouped by years, so we restricted that analysis to 2012‐2016.

### 2.2 Statistical methods

#### 2.2.1 Incidence and death rates

We calculated overall average annual age‐adjusted incidence rates (IR) and death rates (MR) using SEER*Stat 8.3.5.^18^ Average annual rates for 2003‐2016 per 100 000 were age adjusted by the direct method to the 2000 US standard population.^19^ Corresponding 95% confidence intervals (CIs) were calculated, using the Tiwari method, as modified gamma intervals.^20^ To determine differences between subgroups, incidence and death rate ratios (IRR and MRR, respectively) were calculated; rates were considered statistically different if the 95% CIs of the rate ratios excluded one.^21^ Rates and rate ratios were calculated by gender, age group, race/ethnicity, and rural urban status. Individuals younger than 20 years of age were excluded from the denominator.

#### 2.2.2 Trends

Temporal trends in rates were calculated using Joinpoint Regression Program 4.6.00, with a maximum of two joinpoints (up to 3‐line segments) allowed.^22^ Average annual percent change (AAPC) for 2003‐2016 was calculated using weighted average of the slope coefficients of the underlying joinpoint regression line with the weights equal to the length of each segment over the interval. To determine whether the AAPC was statistically different from zero (*P* < .05), a two‐sided *t* test was used for zero joinpoints, and a two‐sided z‐test was used for one or more joinpoints. Rates were considered to increase or decrease if *P* < .05; otherwise rates were considered stable. Trends were calculated by gender, age group, and race/ethnicity.

## 3 RESULTS

### 3.1 Incidence

During 2003‐2016, overall age‐adjusted incidence rates among adults (aged 20 years or older) were 0.45 for solitary plasmacytoma, 0.12 for extramedullary plasmacytoma, and 8.47 for multiple myeloma per 100 000 persons (Table 1). Incidence rates were higher among men than women for solitary plasmacytoma (IRR 1.92, 95% CI 1.85‐1.98), extramedullary plasmacytoma (IRR 1.99, 95% CI 1.86‐2.12), and multiple myeloma (IRR 1.50, 95% CI 1.49‐1.52).

**Table 1:** Average age‐adjusted incidence rates of solitary plasmacytoma, extramedullary plasmacytoma, and multiple myeloma^a^ by gender, age, race/ethnicity^b^, and rural‐urban status^c^, among adults (individuals aged 20 years or older), United States^d^, 2003‐2016

	Plasmacytoma						Multiple Myeloma		
	Solitary			Extramedullary			Total		
	n	IR (95% CI)	IRR (95% CI)	n	IR (95% CI)	IRR (95% CI)	n	IR (95% CI)	IRR (95% CI)
Total^e^	1,066	0.45 (0.44‐0.46)	N/A	297	0.12 (0.12‐0.13)	N/A	20 249	8.47 (8.44‐8.50)	N/A
Gender									
Female	412	0.32 (0.31‐0.32)	1.0 (reference)	111	0.09 (0.08‐0.09)	1.0 (reference)	9170	6.95 (6.91‐6.99)	1.00 (reference)
Male	654	0.61*, a (0.59‐0.62)	1.92 (1.85‐1.98)	184	0.17*, a (0.16‐0.18)	1.99 (1.86‐2.12)	11 079	10.45 (10.40‐10.51)	1.50 (1.49‐1.52)
Age									
20‐49	146	0.12 (0.11‐0.12)	1.0 (reference)	47	0.04 (0.04‐0.04)	1.0 (reference)	1365	1.20 (1.08‐1.11)	1.0 (reference)
50‐59	216	0.52*, a (0.50‐0.54)	4.37 (4.13‐4.63)	57	0.14*, a (0.13‐0.15)	3.56 (3.20‐3.95)	3336	8.00*, a (7.92‐8.07)	7.29 (7.17‐7.41)
60‐69	276	0.97*, a (0.94‐1.00)	8.16 (7.73‐8.61)	78	0.27*, a (0.26‐0.29)	7.07 (6.41‐7.80)	5481	19.25*, a (19.12‐19.39)	17.55 (17.27‐17.83)
70‐79	261	1.51*, a (1.46‐1.56)	12.74 (12.07‐13.46)	67	0.39*, a (0.36‐0.41)	10.01 (9.05‐11.08)	5813	33.84*, a (33.61‐34.08)	30.85 (30.37‐31.34)
≥80	167	1.51*, a (1.45‐1.57)	12.69 (11.95‐13.47)	47	0.42*, a (0.39‐0.46)	10.95 (9.81‐12.22)	4254	38.32*, a (38.02‐38.63)	34.94 (34.37‐35.51)
Race/Ethnicity									
Non‐Hispanic White	767	0.42 (0.41‐0.43)	1.0 (reference)	214	0.12 (0.11‐0.12)	1.0 (reference)	13 936	7.48 (7.44‐7.51)	1.0 (reference)
Non‐Hispanic Black	162	0.67*, a (0.64‐0.70)	1.59 (1.52‐1.67)	39	0.16*, a (0.15‐0.18)	1.39 (1.26‐1.52)	3988	17.43*, a (17.29‐17.58)	2.30 (2.30‐2.35)
Non‐Hispanic American Indian/									
Alaskan Native	—^f^	—	—	—	—	—	104	7.53 (7.13‐7.95)	1.01 (0.95‐1.06)
Non‐Hispanic Asian/Pacific Islander	18	0.18*, a (0.16‐0.20)	0.43 (0.37‐0.48)	—	—	—	471	4.91*, a (4.79‐5.03)	0.66 (0.64‐0.67)
Hispanic	106	0.49*, a (0.47‐0.52)	1.17 (1.10‐1.23)	31	0.14*, a (0.13‐0.16)	1.19(1.06‐1.32)	1599	8.28*, a (8.17‐8.39)	1.11 (1.09‐1.12)
Rural‐Urban Status									
Metropolitan	880	0.46(0.45‐0.46)	1.0 (reference)	245	0.13 (0.12‐0.13)	1.0 (reference)	16 956	8.78 (8.74‐8.82)	1.0 (reference)
Nonmetropolitan	185	0.48*, a (0.46‐0.50)	1.06 (1.02‐1.11)	50	0.13 (0.12‐0.14)	1.02 (0.94‐1.11)	3270	8.24*, a (8.17‐8.32)	0.94 (0.93‐0.95)

Abbreviations: CI, confidence interval; IR, incidence rate; IRR, incidence rate ratio.

^a^ International Classification of Diseases for Oncology, Third Edition (ICD‐O‐3) morphology codes: 9731/3 (solitary plasmacytoma), 9734/3 (extramedullary plasmacytoma), and 9732/3 (multiple myeloma). Rates are per 100,000 persons and age standardized to the 2000 US standard population (19 age groups—Census P25‐1130).

^b^ Racial and ethnic groups are mutually exclusive. Hispanic persons can be any race. Rates are not presented for those with unknown or other race or unknown ethnicity.

^c^ The US Department of Agriculture Economic Research Service 2013 vintage rural‐urban continuum codes were used to categorize county residence at time of cancer diagnosis as metropolitan (codes 1‐3) or nonmetropolitan (codes 4‐9) (https://www.ers.usda.gov/data‐products/rural‐urban‐continuum‐codes).

^d^ Data are compiled from cancer registries that meet data quality criteria, covering 100% of the US population during 2003‐2016.

^e^ Categories may not add to total because of rounding and because unknown values were excluded.

^f^ Data were suppressed when average annual case count < 16.

* Indicates rates were significantly different from zero (*P* < .05).

Age‐specific incidence rates were highest among individuals aged 80 years or older compared to all other age groups with an IR of 1.51 for solitary plasmacytoma, 0.42 for extramedullary plasmacytoma, and 38.32 for multiple myeloma per 100,000 persons. Age‐specific incidence rates were lowest among individuals aged 20‐49 years compared to all other age groups, with an IR of 0.12 for solitary plasmacytoma, 0.04 for extramedullary plasmacytoma, and 1.20 for multiple myeloma per 100,000 persons.

By race/ethnicity, incidence rates were highest among non‐Hispanic blacks compared to all other racial and ethnic groups, with an IR of 0.67 per 100 000 persons for solitary plasmacytoma (IRR compared to non‐Hispanic whites = 1.59, 95% CI 1.52‐1.67), IR = 0.16 for extramedullary plasmacytoma (IRR 1.39, 95% CI 1.26‐1.52), and IR = 17.43 for multiple myeloma (IRR 2.30, 95% CI 2.30‐2.35). Incidence rates of solitary plasmacytoma and multiple myeloma were lowest among non‐Hispanic Asian/Pacific Islander (0.18 and 4.91 per 100,000 persons), whereas incidence rates of extramedullary plasmacytoma were lowest among non‐Hispanic white (0.12 per 100 000 persons).

Compared to individuals living in metropolitan areas, incidence rates of solitary plasmacytoma were slightly higher among individuals living in non‐metropolitan areas (0.48 per 100 000 persons; IRR 1.06, 95% CI 1.02‐1.11); whereas incidence rates of multiple myeloma were lower (8.24 per 100 000 persons; IRR 0.94, 95% CI 0.93‐0.95) and incidence rates of extramedullary plasmacytoma were similar (0.13 per 100 000 persons).

### 3.2 Mortality

Myeloma death rates among adults were 4.47 per 100,000 persons (Table 2). Death rates were statistically significantly higher among men (MR = 5.97) than women (MR = 3.86) with an MRR of 1.55 (95% CI 1.53‐1.56). Compared to individuals aged 20‐49 years (0.26 per 100,000), age‐specific death rates were higher among individuals aged 50‐59 years (RR 10.62, 95% CI 10.28‐10.98), 60‐69 years (RR 32.94, 95% CI 31.94‐33.99), 70‐79 years (RR 79.29, 95% CI 76.92‐81.76), and individuals aged 80 years or older (RR 131.24, 95% CI 127.31‐135.31).

**Table 2:** Average age‐adjusted myeloma death rates^*^, ^a^ by gender, age, race/ethnicity^b^, and rural‐urban status^c^, among adults (individuals aged 20 years or older), United States^d^, 2003‐2016

	n	Rate (95% CI)	RR (95% CI)
Total^e^	11 263	4.77 (4.74‐4.79)	N/A
Gender			
Female	5212	3.86 (3.83‐3.89)	1.0 (reference)
Male	6051	5.97* (5.97‐6.05)	1.55 (1.53‐1.56)
Age (y)			
20‐49	326	0.26 (0.25‐0.27)	1.0 (reference)
50‐59	1158	2.76* (2.72‐2.81)	10.62 (10.28‐10.98)
60‐69	2433	8.57* (8.48‐8.66)	32.94 (31.94‐33.99)
70‐79	3533	20.63* (20.45‐20.82)	79.29 (76.92‐81.76)
≥80	3812	34.15* (33.86‐34.44)	131.24 (127.32‐ 135.31)
Race/Ethnicity			
Non‐Hispanic White	8344	4.44 (4.41‐4.46)	1.0 (reference)
Non‐Hispanic Black	1946	9.12* (9.00‐9.23)	2.05 (2.03‐2.08)
Non‐Hispanic American Indian/Native American	51	4.03* (3.73‐4.36)	0.91 (0.84‐0.98)
Non‐Hispanic Asian/Pacific Islander	206	2.30* (2.22‐2.39)	0.52 (0.50‐0.54)
Hispanic	697	3.97* (3.89‐4.05)	0.89 (0.87‐0.91)
Rural‐Urban Status			
Metropolitan	9971	4.79(4.77‐4.82)	1.0 (reference)
Nonmetropolitan	2075	4.82 (4.77‐4.88)	1.01 (0.99‐1.02)

Abbreviations: CI, confidence interval; R, rate; RR, rate ratio.

^a^ Myeloma deaths were those with International Statistical Classification of Diseases and Related Health Problems, 10th Revision (ICD‐10) codes C90.0 (multiple myeloma) or C90.2 (extramedullary plasmacytoma) as the underlying cause of death. Rates are per 100,000 persons and age standardized to the 2000 US standard population (19 age groups – Census P25‐1130).

^b^ Racial and ethnic groups are mutually exclusive. Hispanic persons can be any race. Rates are not presented for those with unknown or other race or unknown ethnicity.

^c^ The US Department of Agriculture Economic Research Service 2013 vintage rural‐urban continuum codes were used to categorize county residence at time of cancer diagnosis as metropolitan (codes 1‐3) or nonmetropolitan (codes 4‐9) (https://www.ers.usda.gov/data‐products/rural‐urban‐continuum‐codes). Mortality data for rural‐urban status were available for grouped years 2012‐2016.

^d^ Data are from CDC's National Center for Health Statistics National Vital Statistics System

^e^ Categories may not add to total because of rounding and because unknown values were excluded

* Indicates rates were significantly different from zero (*P* < .05).

Myeloma death rates were highest among non‐Hispanic blacks (9.12 per 100 000; RR compared to non‐Hispanic whites = 2.05, 95% CI 2.03‐2.08). Compared to non‐Hispanic whites (MR = 4.44), death rates were lower among non‐Hispanic American Indian/Alaska Natives (4.03 per 100 000; RR 0.91, 95% CI 0.84‐0.98), non‐Hispanic Asian/Pacific Islanders (2.30 per 100 000; RR 0.52, 95% CI 0.50‐0.54), and Hispanics (3.97 per 100 000; RR 0.89, 95% CI 0.87‐0.91).

During 2012‐2016, death rates of individuals living in non‐metropolitan areas (MR = 4.82 per 100 000 persons) were similar to those living in metropolitan areas (MR = 4.79 per 100 000 persons).

### 3.3 Trends

**Figure 1:**
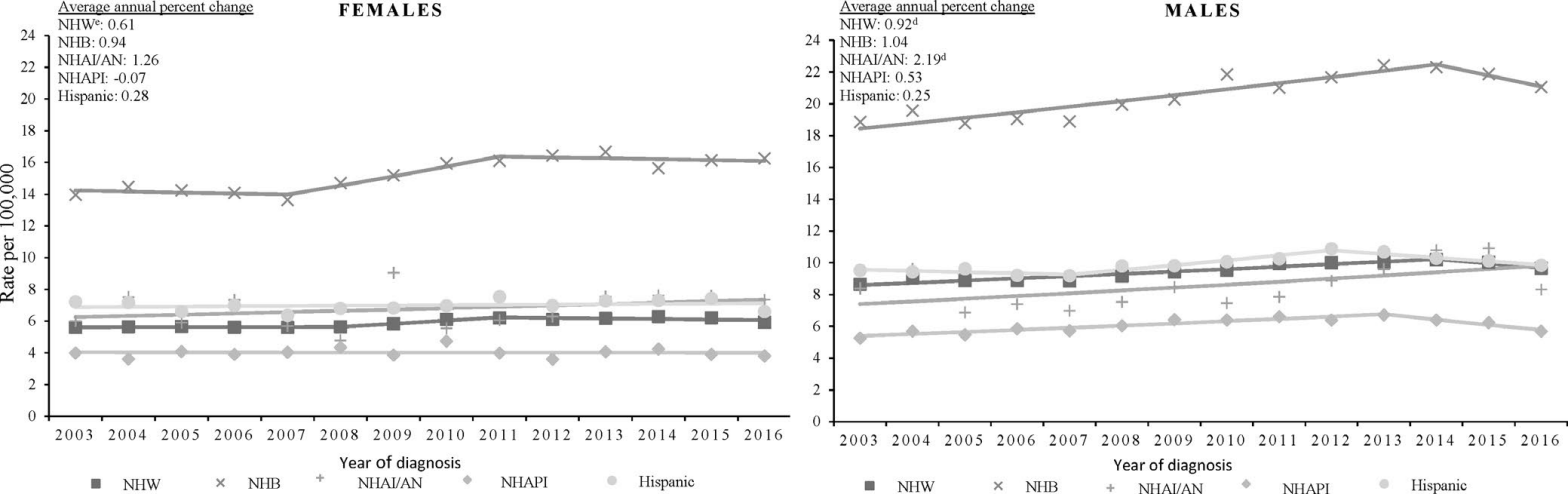
Trends in multiple myeloma age‐adjusted incidence rates^a^ by sex, and race/ethnicity^b^, among adults (individuals aged 20 years or older), United States^c^, 2003‐2016. ^a^Rates are per 100 000 persons and aged standardized to the 2000 US standard population (19 age groups‐ Census P25‐1130) Observed rates are depicted as markers and solid lines depict rates from. ^b^Racial and ethnic groups are manually exclusive. Hispanic persons can be any race. Rates are not presented for those with unknown or other race or unknown ethnicity. ^c^Data are compiled from cancer registries that meet data equality criteria, covering 100% of the US population during 2003‐2016.dIndicates average annual percent change (AACP) significantly differ from zero (*P* < .05). Regression lines and AACP were calculated using join point regression. ^d^NHW = non‐Hispanic White; NHB = non‐Hispanic Black; NHAI/AN = non‐Hispanic American Indian/Alaskan Native; NHAPI = non‐Hispanic Asian/Pacific Islander

Incidence rates of multiple myeloma among men increased 0.81% per year during 2003‐2016 on average. Incidence rates of multiple myeloma among non‐Hispanic white men increased 0.92% per year and among non‐Hispanic American Indian/Alaskan Natives 2.19% per year during 2003‐2016 on average (Figure 1). There were no statistically significant changes among men in any other racial/ethnic group. Incidence of multiple myeloma among women increased 0.75% per year during 2003‐2016 (results did not reach significance). There were no significant changes among women in any racial/ethnic group.

**Figure 2:**
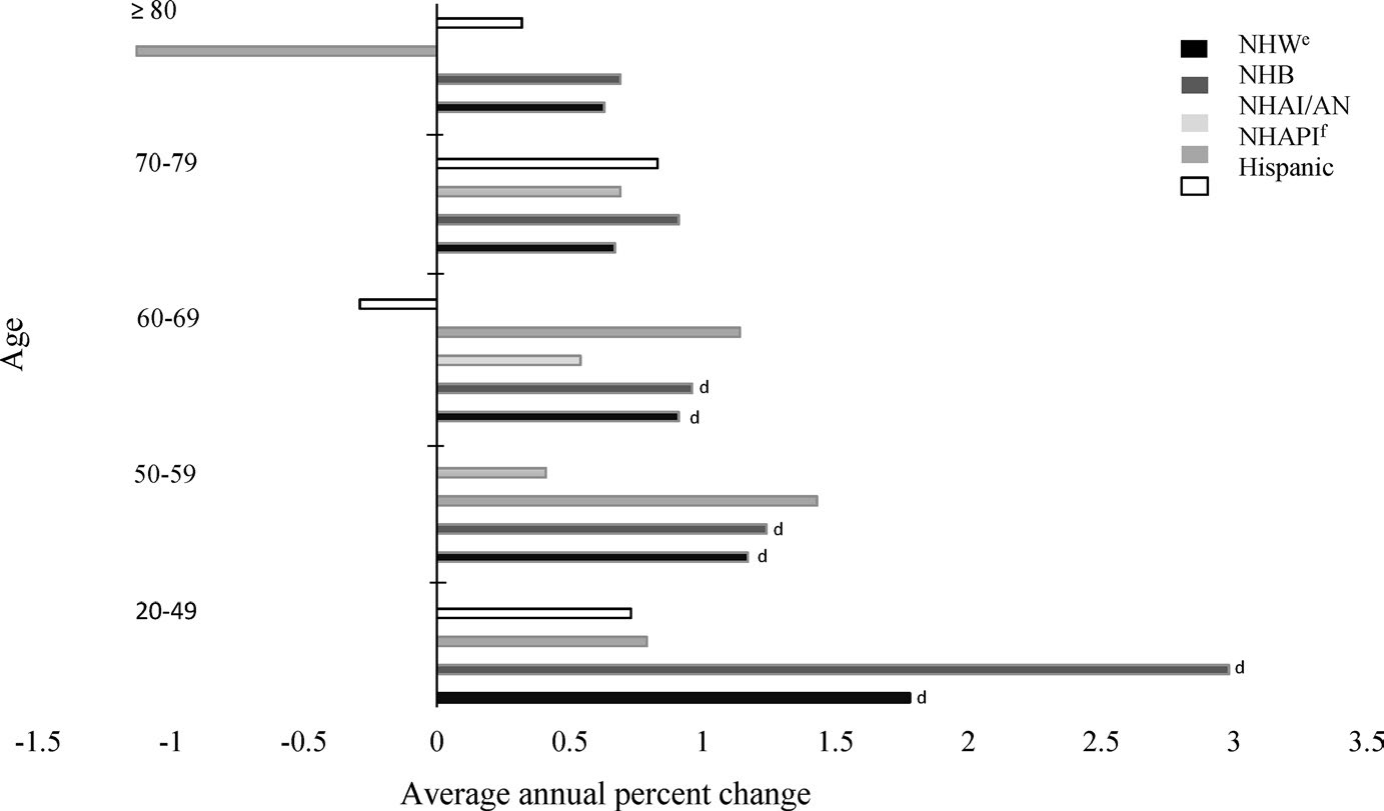
Average annual percent^a^ change in multiple myeloma incidence rates by race/ethnicity^b^, and age, among adults (individuals aged 20 years or older), United States^c^, 2003‐2016. ^a^Average annual percent change (AAPC) was calculated using join point regression. ^b^Racial and ethnic groups are mutually exclusive. Hispanic persons can be any race. Rates are not presented for those with. ^c^Data are compiled from cancer registries that meet data quality criteria covering 100% of the U.S population during 2003‐. ^d^Indicates the AAPC was significantly different from zero (*P* < .05). ^e^NHW = non‐Hispanic White; NHB = non‐Hispanic Black; NHAI/AN = non‐Hispanic American Indian/Alaskan Native; AAPC for APIs was not included for ages 20‐49, 50‐59, 40‐79, and ≥ 80 due to case counts < 16 for certain years

By age groups, incidence rates of multiple myeloma increased 1.78% per year on average for non‐Hispanic whites and 2.98% per year on average for non‐Hispanic blacks 20‐49 years of age; 1.17% per year on average for non‐Hispanic whites and 1.24% per year on average for non‐Hispanic blacks 50‐59 years of age; and 0.91% per year on average for non‐Hispanic whites and 0.96% per year on average for non‐Hispanic blacks 60‐69 years of age (Figure 2).

**Figure 3:**
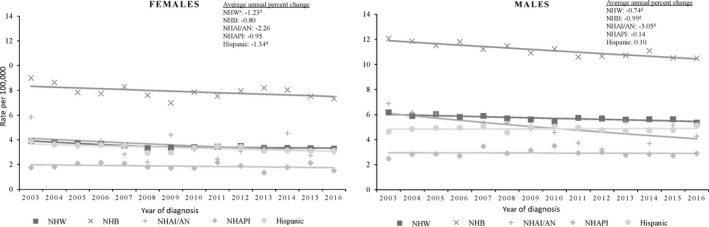
Trends in multiple myeloma age‐adjusted death rates^a^ by sex, and race/ethnicity^b^, among adults (individuals aged 20 years or older), United States^c^, 2003‐2016. ^a^Myeloma deaths were those with International Statistical Classification of Diseases and Related Health Problems, 10th revision (ICD‐10) code C90.0 (multiple myeloma) or C90.2 (extramedullary). ^b^Racial and ethnic groups are mutually exclusive.Hispanic persons can be any race. Rates are not presented for those with unknown or other race or unknown ethnicity. ^c^Data are from CDC’s National Center for Health Statistics National Vital Statistics system. ^d^Indicates average annual percent change (AAPC) significantly different from zero (*P* < .05). Regression lines and AAPC was calculated using joinpoint regression. Observed rates are depicted as markers and solid lines. ^e^NHW = non‐Hispanic White; NHB = non‐Hispanic Black; NHAI/AN = non‐Hispanic American Indian/Alaskan Native, NHAPI = non‐Hispanic Asian/Pacific Islander

During 2003‐2016, myeloma death rates decreased −1.38% for women and −0.77% for men per year on average. Death rates decreased 3.05% per year on average for non‐Hispanic American Indian/Alaska Native men, 0.74% per year on average for non‐Hispanic white men, and 0.99% per year on average for non‐Hispanic black men (Figure 3). Death rates decreased 1.23% per year on average for non‐Hispanic white women and 1.34% per year on average for Hispanic women.

## 4 DISCUSSION

Using national data for US adults (aged 20 years or older), we found that trends in incidence rates for multiple myeloma increased during 2003‐2016 among non‐Hispanic white men, and rates were stable for men in other racial/ethnic groups and for women in all racial/ethnic groups. We also found that myeloma death rates decreased among non‐Hispanic American Indian/Alaska Native, non‐Hispanic white, and non‐Hispanic black men and among non‐Hispanic white and Hispanic women and remained stable in other groups.

The introduction of novel therapeutic agents during the past two decades improved patient outcomes for individuals diagnosed with multiple myeloma.^23^ However, one study based on SEER‐Medicare claims data found substantial variability in therapeutic utilization for multiple myeloma, with apparent inequity for racial‐ethnic minorities.^24^ Additionally, in 2014, the International Myeloma Workgroup introduced new criteria for multiple myeloma allowing practitioners to identify multiple myeloma requiring therapy at an earlier stage.^25^, ^26^ A recent study found that 5‐year relative survival rates improved among non‐Hispanic whites at a magnitude similar to that of non‐Hispanic blacks and Hispanics.^27^ Cancer surveillance data can be used to examine trends in incidence and survival of plasma cell tumors and death attributed to myeloma as a result of changes in diagnostic criteria and treatment with therapeutic agents.

Our study confirmed that, compared to non‐Hispanic whites, non‐Hispanic blacks have the highest incidence rates of solitary plasmacytoma, extramedullary plasmacytoma, and multiple myeloma. Genetic differences may contribute, in part, to the higher incidence seen among black non‐Hispanic.^28^ Compared to whites, blacks have a two‐ to threefold greater prevalence of monoclonal gammopathy of undetermined significance, a precursor of multiple myeloma.^29^, ^30^ Our study found that, compared to non‐Hispanic whites, incidence rates for solitary plasmacytoma and multiple myeloma were lowest among non‐Hispanic Asian/Pacific Islanders. Significantly lower rates of solitary plasmacytoma and multiple myeloma among non‐Hispanic Asian/Pacific Island are consistent with previous findings.^11^

Multiple myeloma is a disease primarily found among older ages with an increase in incidence among older ages.^7^, ^10^ We found that individuals aged 80 years or older had the highest incidence rates of solitary plasmacytoma, extramedullary plasmacytoma, and multiple myeloma, supporting previous findings. One study found the incidence rate of solitary and extramedullary plasmacytoma increased with age, although less markedly than for multiple myeloma.^11^ Advancing age is also associated with increasing incidence of comorbid disorders, which may complicate diagnosis and management of myeloma, leading to poorer prognosis among older myeloma patients.^31^ Adequately addressing individual healthcare needs of the aging population, such as comorbid disorders, physical vulnerability, and access to care may help the prognosis of solitary plasmacytoma, extramedullary plasmacytoma, and multiple myeloma. Comorbidity is associated with the concurrent use of multiple medications and increased risk of drug interactions.^31^ When developing individual treatment plans for older patents, it is important to consider the physical condition and comorbidity status of the patient. ^30^ Assessing an individual's overall condition may help determine their ability to tolerate treatment.^10^

We found that incidence rates of multiple myeloma increased during 2003‐2016 among non‐Hispanic whites and non‐Hispanic blacks aged 20‐69 years, and non‐Hispanic Asian/Pacific Islander aged 60‐69 years. Younger generations are experiencing longer exposure period to adiposity.^32^ Between 1960‐2016, obesity prevalence in the United States increased from 13% to 40% among adults aged 20‐74 years.^33^ During 2015‐2016, it is estimated that 40% of US adults aged 20‐74 have obesity and 32% are overweight.^33^ Multiple myeloma is 1 of 13 cancers that have an association with excess body weight.^34^ A meta‐analysis of five studies reported 15% to 54% higher risk of mortality attributed to multiple myeloma for overweight and obese individuals, respectively.^35^ For women, excess weight in adulthood is associated with increased mortality attributed to multiple myeloma, and effects of obesity may effect both early and late stages of myeloma pathogenesis.^31^ Multifactorial efforts, including communities strategies and clinical interventions, may help prevent and control overweight and obesity and may reduce the risk of cancer.^34^

Our study findings are consistent with other studies showing that multiple myeloma incidence is higher among non‐Hispanic blacks and Hispanics than among non‐Hispanic whites. We also found that multiple myeloma incidence rates increased among those 20‐69 years, but it is still primarily a disease among the elderly. Understanding the role of these characteristics may inform understanding of future myeloma rates. The US population is becoming older; by 2060, it is estimated that about one in five individuals in the United States will be 65 or older.^36^ It is also estimated that by 2044, half of the US population will be from a racial group other than non‐Hispanic white.^36^ Based on expected changes in US demographics, it is predicted that in 2032‐2034, there will be 18,500 new cases of multiple myeloma per year in men (65% increase from 2011) and 13,700 new cases per year in women (61% increase).^37^

A strength of this study is that it uses population‐based data covering the entire US population during 2003‐2016. There are at least three limitations to this study. First, delays in cancer reporting might contribute to an underestimate of recently reported incidence rates. Myeloma is often diagnosed in a nonhospital setting, such as a physician's office; case reporting in these settings is often missed, delayed, or incomplete. To improve case reporting and minimize the burden for physicians, CDC supports efforts to automate reporting from electronic health records using national standards for data exchange, data transmission, and data processing. Second, analyses may be biased if race or ethnicity were misclassified. Reporting of race and ethnicity uses data from medical records and death certificates, which might be inaccurate in some cases.^38^ Methods are used to verify that this race ethnicity data are accurate as possible. To minimize underclassification of Hispanic ethnicity in cancer incidence, cancer registries supported by CDC and NCI assign Hispanic ethnicity through a standardized Hispanic‐Latino identification algorithm. To reduce misclassification of AI/AN race in cancer incidence, selected NCPR registries and all SEER registries link their central cancer registry data with the Indian Health Service (IHS) administrative records database. If a cancer case matches with the IHS database, then race is classified as AI/AN. This helps provide a more comprehensive and accurate picture of the cancer burden in this population. CDC’s National Center for Health Statistics is working with states to improve the reporting of race and ethnicity on death certificates. Third, trend analysis should be carefully interpreted. Even though the AAPC is not statically significant, rates may be increasing or decreasing.

The US population is projected to become older and more racially and ethnically diverse.^8^ It also will have a larger proportion of persons who have had an earlier and longer exposure to excess body weight.^32^ These population changes may impact future trends in incidence of solitary plasmacytoma, extramedullary plasmacytoma, and multiple myeloma, and mortality attributed to myeloma. High‐quality cancer surveillance data allow for routine monitoring of cancer incidence and mortality. Since overweight and obesity increase the risk for multiple myeloma,^34^ the implementation of evidence‐based interventions to prevent and control overweight and obesity may help to reduce new cases of multiple myeloma. Additionally, efforts to adequately address the healthcare needs of the aging population may help reduce incidence of solitary plasmacytoma, extramedullary plasmacytoma, and multiple myeloma and death attributed to myeloma.

## CONFLICT OF INTEREST

The authors declare no potential conflicts of interest.

## AUTHORS’ CONTRIBUTIONS

Taylor D. Ellington: Conception and design, development of methodology, analysis and interpretation of data, writing‐original draft, and writing‐review and editing. S. Jane Henley: Conception and design, development of methodology, analysis and interpretation of data, and writing‐review and editing. Reda J. Wilson: Conception and design, development of methodology, and writing‐review and editing. Manxia Wu: Conception and design, and writing‐review and editing. Lisa C. Richardson: Conception and design, and writing‐review and editing.

## Data Availability

The data that support the findings of this study are available in a restricted access file at the CDC National Center for Health Statistics Research Data Center (https://www.cdc.gov/rdc/B1DataType/Dt131.htm). A public use version of the US Cancer Statistics database is available to researchers at https://www.cdc.gov/cancer/public‐use.

## References

[1] Siegel RL , Miller KD , Jemal A . Cancer statistics, 2015. CA Cancer J Clin, 2015;65(1):5‐29. 25559415 10.3322/caac.21254

[2] Landgren O , Weiss BM . Patterns of monoclonal gammopathy of undetermined significance and multiple myeloma in various ethnic/racial groups: support for genetic factors in pathogenesis. Leukemia. 2009;23:1691‐1697. 19587704 10.1038/leu.2009.134

[3] Thumallapally N , Meshref A , Mousa M , Terjanian T . Solitary plasmacytoma: population‐based analysis of survival trends and effect of various treatment modalities in the USA. BMC Cancer. 2017;17(1):13. 28056880 10.1186/s12885-016-3015-5 PMC5216567

[4] Landgren O , Kyle RA , Pfeiffer RM , et al. Monoclonal gammopathy of undetermined significance (MGUS) consistently precedes multiple myeloma: a prospective study. Blood. 2009;113(22):5412‐5417. 19179464 10.1182/blood-2008-12-194241 PMC2689042

[5] Teras LR , Kitahara CM , Birmann BM , et al. Body size and multiple myeloma mortality: a pooled analysis of 20 prospective studies. Br J Haematol. 2014;166(5):667‐676. 24861847 10.1111/bjh.12935 PMC4134758

[6] Thordardottir M , Lindqvist EK , Lund SH , et al. Obesity and risk of monoclonal gammopathy of undetermined significance and progression to multiple myeloma: a population‐based study. Blood Adv. 2017;1(24):2186‐2192. 29296866 10.1182/bloodadvances.2017007609 PMC5737120

[7] Turesson I , Velez R , Kristinsson SY , Landgren O . Patterns of multiple myeloma during the past 5 decades: stable incidence rates for all age groups in the population but rapidly changing age distribution in the clinic. Mayo Clin Proc. 2010;85(3):225‐230. 20194150 10.4065/mcp.2009.0426 PMC2843108

[8] Sung H , Siegel RL , Rosenber PS , Jemal A . Emergin cancer trends among young adults in the USA: analysis of a population‐based cancer registry. Lancet Public Health. 2019;4:e137‐e147. 30733056 10.1016/S2468-2667(18)30267-6

[9] Kilciksiz S , Karakoyun‐Celik O , Agaoglu FY , Haydaroglu A . A review for solitary plasmacytoma of bone and extramedullary plasmacytoma. Scientific World J. 2012;2012:895765. 10.1100/2012/895765 PMC3354668 22654647

[10] Kyle RA , Therneau TM , Rajkumar SV , Larson DR , Plevak MF , Melton LJ III . Incidence of multiple myeloma in Olmsted County, Minnesota. Trend over 6 decades. Cancer. 2014;101:2667‐2674. 10.1002/cncr.20652 15481060

[11] Dores GM , Landgren O , McGlynn KA , Curtis RE , Linet MS , Devesa SS . Plasmacytoma of bone, extramedullary plasmacytoma, and multiple myeloma: incidence and survival in the United States, 1992–2004. BR J Haematol. 2009;144(1):86‐94. 19016727 10.1111/j.1365-2141.2008.07421.x PMC2610331

[12] Milano AF . Plasma cell myeloma – 20‐year comparative survival and mortality of three plasma cell myeloma ICD‐O‐3 Oncologic phenotypes by age, sex, race, stage, cohort entry time‐period and disease duration: a systematic review of 111,041 cases for diagnosis years 1973–2014: (SEER*Stat 8.3.4). J Ins Med. 2018;47(4):203‐211. 10.17849/insm-47-04-1-9.1 30668210

[13] Waxman AJ , Mink PJ , Devesa SS , et al. Racial disparities in incidence and outcome in multiple myeloma: a population‐based study. Blood. 2010;116(25):5501‐5506. 20823456 10.1182/blood-2010-07-298760 PMC3031400

[14] U.S. Cancer Statistics Working Group . United States Cancer Statistics: 1999–2016. Incidence and mortality Web‐based report. Atlanta, GA: U.S. Department of Health and Human Services, CDC, National Cancer Institute; 2016.

[15] World Health Organization . International classification of diseases for oncology, third edition, first revision. Geneva, Switzerland: World Health Organization; 2014.

[16] National Center for Health Statistics . National Vital Statistics System: Mortality Data. Atlanta, GA: US Department of Health and Human Services, CDC; 2019.

[17] World Health Organization . International statistical classification of diseases and related health problems, tenth revision. World Health Organization. 2015. 3376487

[18] National Cancer Institute . SEER*Stat software. Bethesda, MD: National Cancer Institute, Surveillance Research Program; 2018.

[19] Anderson R , Rosenberg H . Age standardization of death rates: implementation of the year 2000 standard. National Vital Stat Rep. 1998;47:1‐16. 9796247

[20] Tiwari RC , Clegg LX , Zou Z . Efficient interval estimation for age‐adjusted cancer rates. Stat Methods Med Res. 2006;15:547‐569. 17260923 10.1177/0962280206070621

[21] Fay MP . Approximate confidence intervals for rate ratios from directly standardized rates with sparse data. Commun Stat: Theory and Methods. 2007;28(9):2141‐2160.

[22] National Cancer Institute . Joinpoint regression program. Bethesda, MD: National Cancer Institute, Surveillance Research Program, Statistical Methodology and Applications Branch; 2018.

[23] Kumar SK , Dispenzieri A , Lacy MQ , et al. Continued improvement in survival in multiple myeloma: changes in early mortality and outcomes in older patients. Leukemia. 2014;28(5):1122‐1128. 24157580 10.1038/leu.2013.313 PMC4000285

[24] Ailawadhi S , Frank RD , Advani P , et al. Racial disparity in utilization of therapeutic modalities among multiple myeloma patients: a SEER‐medicare analysis. Cancer Med. 2017;6(12):2876‐2885. 29105343 10.1002/cam4.1246 PMC5727310

[25] Rajkumar SV , Dimopoulos MA , Palumbo A , et al. International Myeloma Working Group updated criteria for the diagnosis of multiple myeloma. Lancet Oncol. 2014;15(12):e538‐e548. 25439696 10.1016/S1470-2045(14)70442-5

[26] Landgren O . Shall we treat smoldering multiple myeloma in the near future? Hematology Am Soc Hematol Educ Program. 2017;2017(1):194‐204. 29222256 10.1182/asheducation-2017.1.194 PMC6142564

[27] Costa LJ , Brill IK , Omel J , Godby K , Kumar SK , Brown EE . Recent trends in multiple myeloma incidence and survival by age, race, and ethnicity in the United States. Blood Adv. 2017;1(4):282‐287. 29296944 10.1182/bloodadvances.2016002493 PMC5727774

[28] Smith CJ , Ambs S , Landgren O . Biological determinants of health disparities in multiple myeloma. Blood Cancer J. 2018;8(9):85. 30190459 10.1038/s41408-018-0118-z PMC6127236

[29] Landgren O , Gridley G , Turesson I , et al. Risk of monoclonal gammopathy of undetermined significance (MGUS) and subsequent multiple myeloma among African American and white veterans in the United States. Blood. 2006;107:904‐906. 16210333 10.1182/blood-2005-08-3449 PMC1895893

[30] Landgren O , Katzmann JA , Hsing AW , et al. Prevalence of monoclonal gammopathy of undetermined significance among men in Ghana. Mayo Clin Proc. 2007;82:1468‐1473. 18053453 10.1016/S0025-6196(11)61089-6

[31] Palumbo A , Bringhen S , Ludwig H , et al. Personalized therapy in multiple myeloma according to patient age and vulnerability: a report of the European Myeloma Network (EMN). Blood. 2011;118(17):4519‐4529. 21841166 10.1182/blood-2011-06-358812

[32] Robinson WR , Keyes KM , Utz RL , Martin CL , Yang Y . Birth cohort effects among US‐born adults born in the 1980s: foreshadowing future trends in US obesity prevalence. Int J Obes. 1980;2013(37):488‐554. 10.1038/ijo.2012.66 PMC3448850 22546778

[33] National Center for Health Statistics . Prevalence of overweight, obesity, and severe obesity among adults aged 20 and over: United States 1960–1962 through 2015–2016. Hyattsville, MD: US Department of Health and Human Services, CDC, National Center for Health Statistics; 2018.

[34] Steele CB , Thomas CC , Henley SJ , et al. Vital Signs: Trends in Incidence of Cancers Associated with Overweight and Obesity ‐ United States, 2005–2014. MMWR Morb Mortal Wkly Rep. 2017;66(39):1052‐1058. 28981482 10.15585/mmwr.mm6639e1 PMC5720881

[35] Wallin A , Larsson SC . Body mass index and risk of multiple myeloma: a meta‐analysis of prospective studies. Eur J Cancer. 2011;47:1606‐1615. 21354783 10.1016/j.ejca.2011.01.020

[36] Colby SL , Ortman JM . Projections of the size and composition of the U.S. population: 2014 to 2060. Washington, DC: U.S. Census Bureau; 2015.

[37] Rosenberg PS , Barker KA , Anderson WF . Future distribution of multiple myeloma in the United States by sex, age, and race/ethnicity. Blood. 2015;125(2):410‐412. 25573972 10.1182/blood-2014-10-609461 PMC4287646

[38] Centers for Disease Control and Prevention . U.S. Cancer Statistics: Interpreting Race and Ethnicity in Cancer Data. Atlanta, GA: US Department of Health and Human Services, CDC; 2019.

